# Production of the sheep pox virus structural protein SPPV117 in tobacco chloroplasts

**DOI:** 10.1007/s10529-021-03117-x

**Published:** 2021-04-02

**Authors:** Gulshan Stanbekova, Daniyar Beisenov, Anna Nizkorodova, Bulat Iskakov, Heribert Warzecha

**Affiliations:** 1 Protein and Nucleic Acids Research, M. Aitkhozhin Institute of Molecular Biology and Biochemistry, Almaty, Kazakhstan; 2 Plant Biotechnology and Metabolic Engineering, Technical University of Darmstadt, Darmstadt, Germany

**Keywords:** Sheep pox virus, SPPV117 protein, Transplastomic plants

## Abstract

**Objective:**

A chloroplast transgenic approach was assessed in order to produce a structural protein SPPV117 of sheep pox virus in *Nicotiana tabacum* for the future development of a plant-based subunit vaccine against sheep pox.

**Results:**

Two DNA constructs containing SPPV117 coding sequence under the control of chloroplast promoter and terminator of *psbA* gene or *rrn* promoter and *rbcL* terminator were designed and inserted into the chloroplast genome by a biolistic method. The transgenic plants were selected via PCR analysis. Northern and Western blot analysis showed expression of the transgene at transcriptional and translational levels, respectively. The recombinant protein accumulated to about 0.3% and 0.9% of total soluble protein in leaves when expressed from *psbA* and *rrn* promoter, respectively. Plant-produced SPPV117 protein was purified using metal affinity chromatography and the protein yield was 19.67  ±  1.25 µg g^−1^ (FW)_._ The serum of a sheep infected with the virus recognised the chloroplast-produced protein indicating that the protein retains its antigenic properties.

**Conclusions:**

These results demonstrate that chloroplasts are a suitable system for the production of a candidate subunit vaccine against sheep pox.

**Supplementary Information:**

The online version contains supplementary material available at 10.1007/s10529-021-03117-x.

## Introduction

Sheep pox is a highly contagious disease of small ruminants, which has a wide global distribution area. It causes large economic losses in livestock animals; therefore, it is classified as a notifiable disease by the World Organization for Animal Health (https://www.oie.int/animal-health-in-the-world/oie-listed-diseases-2020). The causative agent is sheep pox virus (SPPV), a member of the genus *Capripoxvirus* within the Poxviridae family. Currently, for the prevention of sheep pox, attenuated SPPV strains are widely used. However, live attenuated virus vaccines are potentially dangerous and can revert to virulent forms, causing disease. Therefore, alternative vaccination strategies based on recombinant, immunogenic proteins of the pathogen are sought after. Plants represent an attractive system for recombinant vaccine production because of their ease of scalability, low cost of production compared to other eukaryotic systems such as yeast and animal cells, and the absence of human and animal pathogens in the production host (Fischer and Buyel 2020). Plants produce and process eukaryotic proteins much better than can bacteria or even yeasts (Rybicki 2018). Producing recombinant proteins in plant chloroplasts has several advantages over nuclear integration of the transgene including an absence of gene silencing and position effects. Another advantage is a high expression level of a target gene. Here, the content of recombinant proteins in transplastomic plants can reach more than 70% of total soluble protein (TSP) (Castiglia et al. 2016). A number of antigens have been successfully produced in chloroplasts, including antigens of dengue virus, poliovirus, and *Mycobacterium tuberculosis* (van Eerde et al. 2019; Daniell et al. 2019; Saba et al. 2019), providing the rationale for producing a plant-based, low-cost animal vaccine against sheep pox.

A 17-kDa protein encoded by the *sppv117* gene (Tulman et al. 2002) is an ortholog of the A27L protein of vaccinia virus, the most investigated poxvirus to date. A27L is an envelope protein located in the intracellular mature virion (IMV) membrane and plays a major role in virus penetration (Rodriguez et al. 1987). The fact that bacterially produced SPPV117 protein induced virus-neutralising antibodies to SPPV in laboratory animals (Chervyakova et al. 2016) supported the notion that this plant-produced protein will also retain high immunogenicity. In this study, we generated and characterised transplastomic tobacco plants expressing the candidate vaccine protein SPPV117.

## Materials and methods

### Protein sequence analysis and in silico epitope prediction

The amino acid sequence of SPPV117 (GenBank ID: NP_659689) was analysed for the presence of putative transmembrane domains, signal peptides and N-, O-, and C-linked glycosylation sites using TMHMM v. 2.0 (Krogh et al. 2001), SignalP v. 5.0 (Almagro Armenteros et al. 2019), NetNGlyc v. 1.0 (Gupta and Brunak 2002), NetCGlyc v. 1.0 (Julenius 2007), and NetOGlyc v. 4.0 (Steentoft et al. 2013) tools, respectively. B-cell epitope identification was performed using the AAPPred method (Davydov and Tonevitsky 2009) and Bepipred v. 2.0 method (Jespersen et al. 2017), setting threshold at 0.55 and a peptide length > 6. NetMHCpan v. 4.0 and NetMHCIIpan v. 4.0 algorithms were used to predict T-cell MHC I and MHC II epitopes. The SPPV117 protein sequence was analysed against 32 MHC I and 37 MHC II alleles from sheep, available in the Immuno Polymorphism Database (IPD) (https://www.ebi.ac.uk/ipd/mhc/). Peptides with a length of nine amino acids and a probability score > 0.55 and peptides with a length of 15 amino acids and a score > 0.7 were considered potential MHC I and MHC II antigens, respectively. All programs except AAPPred are available at http://www.cbs.dtu.dk/services/. AAPPred method is available at https://www.bioinf.ru/.

### Plasmid construction

Previously, we cloned the *sppv117* gene as a part of a vector pl/his:sppv117 (Beisenov et al. 2016). The 539 bp purified NcoI-XbaI fragment containing *his*_*10*_*:sppv117* fusion gene from the plasmid pl/his:sppv117 was cloned into the vector pkG27 (Glenz et al. 2006), replacing the *uidA* gene and generating a plasmid pKG27/his:sppv117. In parallel, the same fragment was blunted at the NcoI site and subcloned into the vector pHK20 (Kuroda and Maliga 2001), which was cut with NdeI and XbaI and blunted on the NdeI site. The resulting plasmid pHK20/his:sppv117 contained the *his*_*10*_*:sppv117* fusion gene instead of the *neo* gene*.* The 1002 bp and 905 bp purified fragments from pKG27/his:sppv117 and pHK20/his:sppv117 plasmids comprising the whole expression cassettes between the HindIII and SacI sites were cloned into the pNT4 vector, a derivative of pRB95 (Ruf et al. 2001), resulting in the final vectors pP117 (pNT4/PpsbA-his:sppv117-TpsbA) and pR117 (pNT4/Prrn-his:sppv117-TrbcL).

### Chloroplast transformation

*Nicotiana tabacum* (cv. Petit Havana) leaves from plants grown under aseptic conditions on Murashige-Skoog (MS) medium were bombarded with a PDS-1000/He Biolistic Particle Delivery System (Bio-Rad, Hercules, CA, USA). For regeneration, leaf explants were placed on RMOP regeneration medium (Svab et al. 1990) containing 500 mg L^−1^ spectinomycin dihydrochloride. The plates were incubated at 23 °C under a 16 h/8 h (light/dark) cycle. Regenerated shoots were rooted on MS medium with 500 mg L^−1^ spectinomycin. Genomic DNA was isolated from the leaves of putative transplastomic plants using the DNeasy Plant Mini Kit (Qiagen, Maryland, USA) according to the manufacturer’s instructions. Integration of *sppv117* and *aadA* genes into the plant genome was confirmed by PCR with two pairs of primers (sppv117-for: 5′-GCATCATATGGACAGAGCGTTATCAATCTTTCCAGGCGA-3′ and sppv117-rev: 5′-GCATCTCGAGTCACTTTAGTGTTGTAATTCTTCCTGTTT-3′; aadA-for: 5′-ATGGCAGAAGCGGTGAT-3′ and aadA-rev: 5′-TTATTTGCCGACTACCTTGGTG-3′). The reaction was carried out in the following temperature conditions: 94 °C for 5 min and 30 cycles of 94 °C for 30 s, 54 °C for 30 s and 72 °C for 1 min; final extension 72 °C for 5 min.

### Measurement of pigment content

Three mature leaves from three individual plants per construct were tested. Leaf samples (0.1 g) were homogenised with 2 mL of ice-cold 96% ethanol and extracts were centrifuged at 10,000×*g* for 15 min at 4 °C. Then 0.5 mL of the supernatant was mixed with 4.5 mL of 96% ethanol and the optical densities were measured at 664, 649 and 470 nm on an Ultrospec 2000 spectrophotometer (Pharmacia Biotech, Cambridge, England). The pigment concentration was calculated according to a previous study (Lichtenthaler 1987).

### Southern blot analysis

After digestion with EcoO109I, tobacco DNA samples (2 μg) were separated on a 0.8% agarose gel, transferred by capillary blotting to a positively charged nylon membrane (Roche, Mannheim, Germany) and hybridised with DIG-labelled probes. Probes were amplified with primer pairs tfr-for/tfr-rev for *trnG/trnfM* region (5′-CGACGGAGAGGGGGTCCACC-3′ and 5′-GAAGCCCCTTTACCATTCTGTAT-3′) from the template of wild-type DNA; sppv117-for/sppv117-rev for *sppv117* gene from plasmid pKG27/his:sppv117; aadA-for/aadA-rev for *aadA* gene from plasmid pNT4 using the PCR-DIG Probe Synthesis Kit (Roche, Mannheim, Germany). Hybridisation was carried out at 42 °C overnight. DNA hybrids were detected with Anti-Digoxigenin-AP Fab fragments (Roche, Mannheim, Germany) at a 1:5000 dilution. Detection was performed using the chemiluminescent substrate CSPD (Roche, Manheim, Germany).

### Northern blot analysis

RNA was extracted using Trizol reagent (Sigma-Aldrich, St. Louis, USA) according to the manufacturer’s instructions. RNA samples (5 μg) were separated on a 1.2% agarose gel containing formaldehyde, transferred to a nylon membrane (Roche, Manheim, Germany) and subsequently incubated with the *sppv117* probe. Hybridisation was carried out at 50 °C overnight. RNA-DNA hybrids were detected as described for Southern blotting.

### qPCR

Three plants per construct and three replicates per plant were analysed. Untransformed plants served as controls. Prior to the reverse transcription RNA samples were treated with TURBO DNA-free Kit (Invitrogen, Carlsbad, CA, USA) according to the manufacturer’s protocol. One microgram of total RNA was used for cDNA-synthesis according to the manufacturer’s protocol, using the SuperScript IV RT (Thermo Fisher Scientific, Vilnius, Lithuania). Primers for target gene were qsppv117-for (5′-ATGGACAGAGCGTTATCAATCTTTCCA-3′) and qsppv117-rev (5′-ATTGGGTTCTTCATCGCTTAATTCCA-3′). The *Nicotiana tabacum rem1* (GenBank: KJ808744) was used as a reference gene. Primers for its detection were rem-for (5′-GCCTCCTCCTGCAGAAGAAA-3′) and rem-rev (5′-CGAGCAAGCACAGCATCTCT-3′). qPCR chain reactions were carried out on QuantStudio 5 (Applied Biosystems, Foster City, USA) using a Fast SYBR Green Master Mix (Applied Biosystems, Foster City, USA). PCR cycling conditions were as follows: 95 °C for 5 min, 40 cycles at 95 °C for 15 s, 58 °C for 60 s, and a final melting curve between 60 and 99 °C (Δ1 °C/s). Relative gene expression level was calculated using Q-Gene program (Muller et al. 2002).

### Western blot analysis

Total soluble protein (TSP) extracts were obtained by homogenisation of 100 mg of leaf samples in 200 µL of extraction buffer (137 mM NaCl, 10 mM Na_2_HPO_4_, 1.8 mM KH_2_PO_4_, 2.7 mM KCl, 0.1% Triton X-100 (v/v), 2 mM phenylmethylsulphonyl fluoride (PMSF), pH 7.4). Lysates were clarified by centrifugation at 12,000×*g* for 10 min at 4 °C. Protein quantification was performed using the Bradford assay (Sigma-Aldrich, St. Louis, USA) by comparing samples relative to known concentrations of bovine serum albumin (BSA). Protein samples (5 µg) were separated by 15% SDS-PAGE gel and transferred to a PVDF membrane (Bio-Rad, USA). For SPPV117 detection, protein-specific polyclonal rabbit antisera (Research Institute for Biological Safety Problems, Kazakhstan) were used as primary antibodies at a 1:2000 dilution. Anti-rabbit IgG conjugated with horseradish peroxidase (Santa Cruz Biotechnology, Heidelberg, Germany) was used as the secondary antibody at a 1:5000 dilution. Detection was performed using the Chemiluminescent Peroxidase Substrate-3 (Sigma-Aldrich, St. Louis, USA). Quantification of SPPV117 in transplastomic plants by densitometry of blots was performed using Image J software (NIH, Bethesda, USA). Experiments were repeated three times. For purified proteins, murine penta-his antibodies and anti-mouse IgG conjugated with horseradish peroxidase (5-Prime, Hamburg, Germany) were used as primary and secondary antibodies, respectively at a 1:6000 dilution. Detection was performed using the Metal Enhanced DAB Substrate Kit (Thermo Scientific, Rockford, USA). Antigenicity of SPPV117 protein was detected using the serum from a sheep infected with sheep pox virus (Research Institute for Biological Safety Problems, Kazakhstan) at a 1:200 dilution. Anti-sheep IgG conjugated with alkaline phosphatase were used as secondary antibodies at a 1:2000 dilution. Alkaline phosphatase activity was detected by the Fast Red TR/Naphthol AS MX substrate (Sigma-Aldrich, St. Louis, USA).

### Purification of His-tagged proteins

SPPV117 protein purification was performed three times under native conditions using 10 g of plant material. Leaves were ground in liquid nitrogen with a mortar and pestle in extraction buffer (50 mM NaH_2_PO_4_, 0.3 M NaCl, 2 mM imidazole, 0.1% Triton X-100 (v/v), 15 mM β-mercaptoethanol, 2 mM PMSF, pH 8.0). The lysate was clarified by centrifugation at 10,000×*g* for 20 min at 4 °C. The supernatant was passed through a 0.45-µm filter and was mixed with PerfectPro Ni-NTA agarose (5-Prime, Hamburg, Germany). The mixture was agitated at 4 °C for 1 h, transferred to a column and allowed to drain under gravity. The column was washed twice with washing buffer (50 mM NaH_2_PO_4_, 0.3 M NaCl, 2 mM imidazole, pH 8.0). The protein bound to the agarose was eluted with elution buffer (50 mM NaH_2_PO_4_, 0.3 M NaCl, 250 mM imidazole, pH 8.0) and collected as 1-mL fractions. The purification process was repeated. Eluted fractions were subjected to Western blot and the protein-containing fractions were concentrated by ultrafiltration through a 3000 MWCO HY column (Amicon, Carrigtwahill, Co. Cork, Ireland). The protein concentration was measured using Bradford reagent (Sigma-Aldrich, St. Louis, USA).

## Results

### Protein sequence analysis and in silico epitope prediction

Viral envelope proteins frequently contain glycans and it has been shown that the removal of the glycan residues may enhance, interfere with, or not affect their immunogenicity (Gavrilov et al. 2011). Recombinant proteins synthesized in chloroplasts can undergo posttranslational modifications, including disulfide bond formation and lipid modifications but no glycosylation (Arlen et al. 2007).

To assess if SPPV117 is a potential glycoprotein, the protein sequence was analysed for the presence of putative glycosylation sites using the NetNGlyc, NetCGlyc, and NetOGlyc predictive tools. The analysis showed the presence of four potential sites for O-linked glycosylation (Ser_6_, Thr_15_, Thr_26_, and Ser_27_) in SPPV117. In silico epitope prediction is used in vaccine development broadly, so we analysed the SPPV117 sequence for potential B- and T-cell epitopes. Prior to searching for B-cell epitopes, the protein sequence was analysed for the presence of transmembrane domains and signal peptides to exclude them from prediction. TMHMM and SignalP tools showed the absence of any transmembrane domains and signal peptides in the protein. For the identification of potential linear B-cell epitopes, two methods were utilised. Bepipred-2.0 algorithm, trained on epitopes determined from 3D structures, predicted two peptides in SPPV117. The peptide sequences ranging from 9–45 and 61–71 amino acid residues can provoke the B-cell immune response. As predicted by AAPPred method, based on amino acids pair frequencies and amino acids scales, three regions 26–39, 46–54, and 112–121 are denoted as potential antigens. By comparing the foregoing results, the peptide sequences indicated in Table 1 were selected as promising linear B-cell epitopes. The region 9–45 is located at the *N*-terminus of the protein that contains putative glycosylation sites. Taking into account the fact that the bacterially expressed SPPV117 protein induced the production of virus-neutralizing antibodies (Chervyakova et al. 2016), this region probably retained its antigenic and immunogenic properties in the absence of glycosylation. Alternatively, other peptides predicted for the central and *C*-terminal protein part are true linear B-cell epitopes in vivo. To define the probability of T-cell epitopes, NetMHCpan and NetMHCIIpan analysis tools were used. As shown in Table 1, the SPPV117 was predicted to have two MHC I and three MHC II epitopes defined in a sheep system. These prediction results suggest that the SPPV117 protein could manifest protective immunity against SPPV.

**Table 1:** Prediction of linear B-cell and T-cell epitopes from the SPPV117 protein sequence

Epitope set	Peptide	Epitope region
B-cell	PGDDDETNERNINHREKTSNDHGHYEDNLLELSDEEP^a^	9–45
	NMIKIKNDI^b^	46–54
	RYSNYISIND^a^	61–71
	SLKNIIKRLE^b^	112–121
T-cell MHC I	DSFISNEEI	79–87
	RLENHIETI	119–127
T-cell MHC II	YSNYISINDDEISNI	62–76
	LKDSFISNEEIQIKD	77–91
	RKNMIVLTKKVDFQT	128–142

^a,b^B-cell epitope prediction was made using Bepipred-2.0 and AAPPred methods, respectively. NetMHCpan-4.0 and NetMHCIIpan-4.0 algorithms were used for prediction of T-cell MHC I and MHC II epitopes

### Expression constructs and tobacco chloroplast transformation

**Fig. 1:**
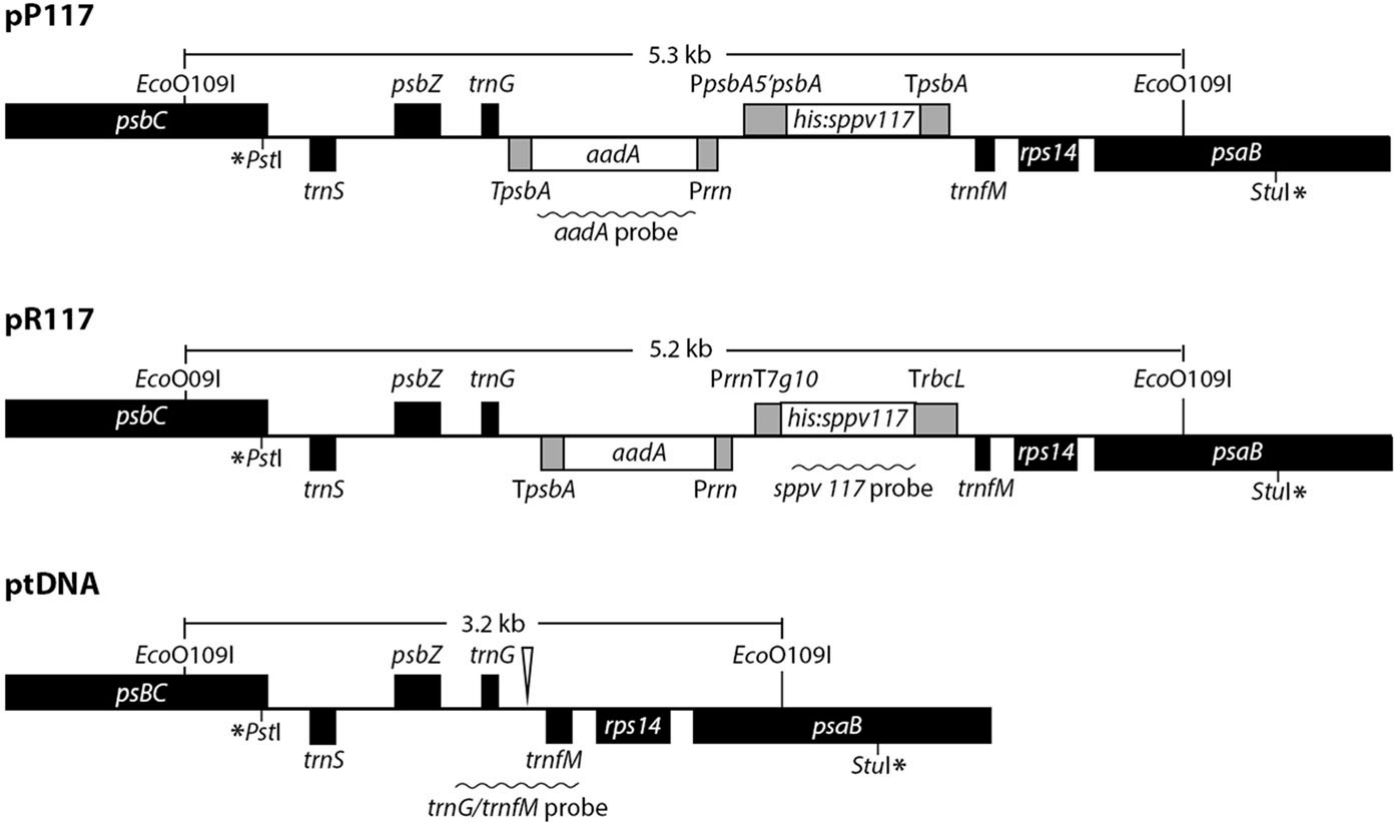
Chloroplast transformation vectors pP117 and pR117. In pP117, a sequence encoding ten histidine residues fused with *sppv117* gene (his:sppv117) was placed between *psbA* promoter (P*psbA*) with 5′-UTR (5′*psbA*) and *psbA* terminator (T*psbA*). In pR117, *his:sppv117* was inserted between the *rrn* promoter with T7*g10* leader (P*rrn*T7*g10*) and *rbcL* terminator (T*rbcL*). Both vectors contain the *aadA* selectable marker gene under the control of the *rrn* promoter and *psbA* terminator. Black boxes depict tobacco chloroplast genes. Vector borders are marked by asterisks. Genes above the line are transcribed from left to right; genes below the line are transcribed from right to left. The sizes of expected EcoO109I-fragments in transformed and non-transformed (ptDNA) chloroplast genome are indicated. Wavy lines indicate the locations of the RFLP probes. The triangle indicates the site of the transgene embedding

To produce SPPV117 protein in tobacco plastids, two expression constructs were designed. One was designed to examine the expression of the *sppv117* gene under the control of the promoter with a 5′-untranslated region (5′**-**UTR) and terminator of the plastid *psbA* gene coding for the photosystem II protein D1 (pP117). The second construct contained the viral gene between the promoter of the tobacco plastid ribosomal RNA operon (P*rrn* with the leader sequence of gene *10* of phage T7) and the terminator of the plastid *rbcL* gene coding the large subunit of ribulose bisphosphate carboxylase (pR117). Both constructs were cloned into the pNT4 vector, whose flanking sequences ensure transgene integration by homologous recombination between the *trnG* and *trnfM* genes in the large single-copy (LSC) region of the chloroplast genome. This vector contains the *aadA* gene encoding aminoglycoside adenylyltransferase to confer resistance to the antibiotics spectinomycin and streptomycin. The *aadA* gene is driven by the *rrn* promoter and *psbA* terminator. Figure 1 presents the resulting transformation vectors pP117 and pR117.

Tobacco leaves were transformed by particle bombardment using both plastid vectors. As a result, after 6–9 weeks, several spectinomycin-resistant shoots were obtained for each construct. All putative transformants were analysed for transgene insertion by PCR of genomic DNA with gene-specific primers (Supplementary Fig. 1). PCR analysis confirmed the transgenic nature of seven shoots and excluded spectinomycin-resistant spontaneous mutants. For further analysis, six plants (three plants per construct) were selected. To eliminate wild-type plastids and achieve a homoplasmic state the obtained tobacco lines were subjected to four additional rounds of regeneration on spectinomycin-containing selective medium, and after such selection were further analysed.

### Characterisation of transplastomic plants

The transplastomic plants generated from the vector pP117 (P117 lines) were phenotypically indistinguishable from wild-type plants, while all plants transformed with pR117 (R117 lines) showed signs of stunted growth and a pale-green leaf colour (Supplementary Fig. 2). Despite this, they developed normally and set seeds upon self-pollination. A pale leaf colour indicates a change in pigment content, hence the content of pigments in the transplastomic lines and wild-type plants was determined. The results are shown in Table 2. The pigment content in P117 lines was similar to wild-type plants, while in R117 lines, the content of chlorophyll was reduced by about 46%. The content of carotenoids in all transformed lines was approximately the same and did not differ from wild type.

**Table 2:** Pigment content in transplastomic plants (mg g^−1^ of fresh weigh)

Pigment	Wild-type	P117 plants	R117 plants
Chlorophyll *a*	1.70 ± 0.04	1.65 ± 0.07	1.03* ± 0.04
Chlorophyll *b*	0.74 ± 0.07	0.72 ± 0.06	0.29* ± 0.08
Total chlorophylls	2.44 ± 0.09	2.36 ± 0.08	1.31* ± 0.06
Total carotenoids	0.33 ± 0.03	0.29 ± 0.04	0.30 ± 0.01

Values are expressed as mean ± SD (n = 9). All measurements were performed in three biological replicates. Values with significant difference from the wild-type control marked with an asterisk (P ≤ 0.001)

### Molecular analysis of plants

**Fig. 2:**
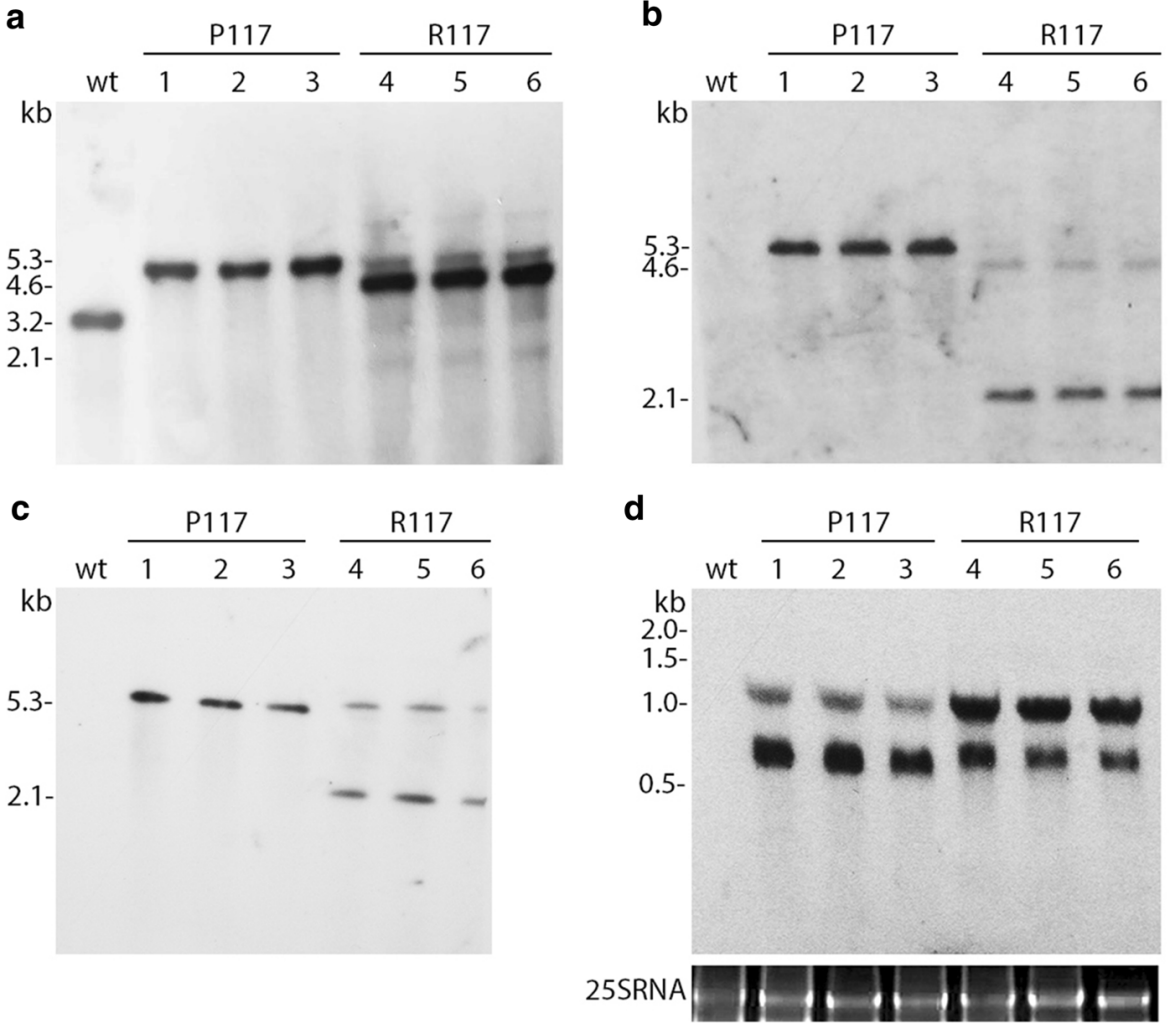
Restriction fragment length polymorphism (RFLP) analysis of P117 and R117 plants. **a**, **b**, **c** Southern blot analysis with probes specific for *trnG/trnM*, *sppv117* and *aadA* genes, respectively. **d** Northern blot analysis of total RNA with *sppv117* probe. The lower gel stained with ethidium bromide demonstrates the amount of analysed RNA. *wt* wild-type tobacco plants; 1–6, analysed plants

Restriction fragment length polymorphism (RFLP) analysis was performed to demonstrate the site-specific integration of the transgene as well as the homoplasmy of the transformed plants. Total DNA isolated from plants was digested with EcoO109I that cut outside of the flanking recombination regions and probed with a 0.7 kb fragment comprising the transgene insertion site *trnG/trnfM* with adjacent sequences of the chloroplast genome (Fig. 1). The probe hybridised with a 3.2 kb DNA-fragment from wild-type plants (Fig. 2a), whereas DNA from P117 lines gave a single band with an expected size of 5.3 kb (Fig. 2a, lanes 1–3). In R117 lines, in addition to the predicted fragment of 5.2 kb, several additional bands including the putative wild-type 3.2 kb fragment were observed (Fig. 2a, lanes 4–6). A 4.6 kb fragment gave a significantly more intense signal than the expected 5.2 kb fragment. Hybridisation with a 0.5 kb probe specific for *sppv117* gene identified a single strong expected 5.3 kb band in P117 plants (Fig. 2b, lanes 1–3), while in DNA from R117 plants there were a weak expected 5.2 kb band and two additional bands of 4.6 kb and 2.1 kb (Fig. 2b, lanes 4–6). A 0.8 kb *aadA* probe gave a single predicted 5.3 kb band in P117 lines (Fig. 2c, lanes 1–3), whereas in R117 lines it gave two bands of 5.2 kb and 2.1 kb (Fig. 2c, lanes 4–6). Southern blot analysis demonstrated the homoplasmic state of P117 plants. The detection of unexpected bands in R117 lines suggest that rearrangements occurred in the chloroplasts after transformation, but additional experiments are needed to clarify their nature.

Furthermore, we determined the transgene expression level by Northern blot analysis. Hybridisation of total cellular RNA samples with a gene-specific probe showed that the *sppv117* gene was efficiently transcribed as two specific transcripts in all analysed lines (Fig. 2d). Along with the monocistronic transcript (0.74 kb for P117 lines and 0.81 kb for R117 lines), a longer product (~ 1.3 kb) was detected, apparently due to ineffective transcription termination, which is frequently observed in plastids (Stem and Gruissem 1987). Moreover, monocistronic RNA prevailed in the P117 plants, in contrast to the R117 plants in which a longer product dominated. Performed qPCR-analysis revealed no difference in *sppv117* expression level between P117 and R117 transplastomic lines (Supplementary Fig. 3).

### Protein expression and purification

**Fig. 3:**
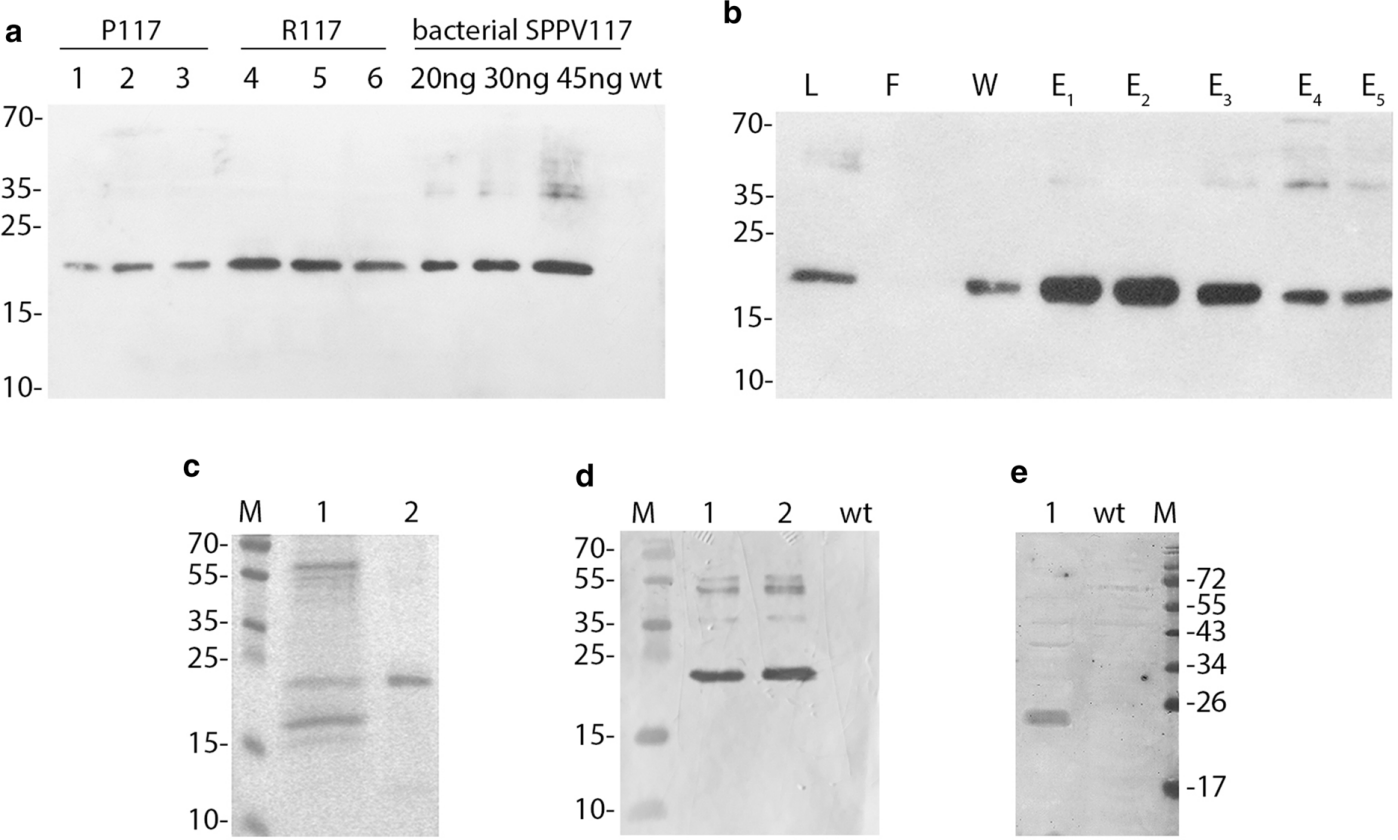
Analysis of SPPV117 protein accumulation in leaves from P117 and R117 plants. **a** Western blot analysis of total soluble proteins from transplastomic (lanes 1–6) with SPPV117-specific antibodies. Different amounts of the bacterially produced protein were used for protein quantitation. Plant protein samples were loaded at 5 µg per lane. **b** Western blot using penta-his antibodies to detect SPPV117 in Ni–NTA fractions from R117 plants. *L* lysate, *F* flow throw fraction, *W* wash fraction, *E*_*1*_–*E*_*5*_, eluted fractions. **c**, **d** Coomassie blue-stained SDS-PAGE and Western blot using SPPV117-specific antibodies after first (lane 1) and second (lane 2) rounds of chromatography. **e** Western blot of purified chloroplast-produced SPPV117 protein (0.5 µg, lane 1) with serum from a sheep infected with sheep pox virus. Molecular mass marker (M) sizes are indicated in kDa. Protein samples (5 µg) from untransformed plants (wt) contained in the same buffer as proteins from transplastomic lines were used as negative control

Accumulation of the recombinant SPPV117 protein in transplastomic plants was determined by immunoblotting using antibodies against bacterially synthesised SPPV117. Figure 3a shows the detection of an approximately 20-kDa protein in all analysed lines. Moreover, the SPPV117 protein content was slightly lower in P117 lines expressing the protein from the *psbA* promoter. A comparative densitometric analysis of protein band intensities relative to known amounts of purified bacterially synthesised SPPV117 showed that accumulation of the recombinant protein reached ~ 0.3% of TSP when expressed from the *psbA* promoter and ~ 0.9% of TSP when expressed from the *rrn* promoter.

SPPV117 was purified from R117 lines as they produced the protein at a higher level. The chloroplast-produced protein, fused at the *N*-terminus with deca-histidine, was purified from leaf material of three R117 lines separately by metal affinity chromatography on nickel-nitrilotriacetic acid (Ni-NTA) agarose and analysed by immunoblotting (Fig. 3b). The SPPV117-containing fractions were pooled; their purity was verified by Coomassie blue staining after SDS-PAGE. SDS-PAGE revealed a significant proportion of endogenous proteins (Fig. 3c, lane 1). Therefore, the mixture was subjected to the second round of affinity chromatography followed by ultrafiltration for protein concentration. After a two-step procedure, a protein sample with a purity of about 90% was obtained (Fig. 3c, lane 2). A subsequent immunoblot analysis with his-tag antibodies revealed a 20 kDa protein corresponding to the SPPV117, as well as a small amount of proteins with a higher molecular mass, which presumably correspond to oligomers of SPPV117 (Fig. 3d). The yield of the purified recombinant SPPV117 protein from individual R117 lines, estimated by Bradford assay, was 19.67 ± 1.25 μg g^−1^ of fresh leaf weight. Western blotting was also performed using the serum from a sheep infected with sheep pox virus (Fig. 3e). Convalescent sheep serum recognised SPPV117 purified from plants, indicating that the chloroplast-produced protein retains its antigenic properties.

## Discussion

Plants can be a cheap source of immunogenic antigens of human and animal pathogens for the production of next generation vaccines. In our previous work, we produced transgenic tobacco plants in which the expression level of SPPV117 protein varied from 0.01 to 0.03% of TSP (Beisenov et al. 2016). In the present study, in order to obtain higher yields, we targeted the viral gene to the chloroplast genome. Two DNA constructs containing the SPPV117 coding sequence under the control of different plastid regulatory signals were inserted between the *trnG* and *trnfM* genes in the LSC region of the chloroplast genome. Accumulation of SPPV117 protein was estimated as 0.3% and 0.9% of the TSP when expressed from the *psbA* and *rrn* promoters, respectively. The differences at the expression level between the lines can be attributed to the different 5′- and 3′-UTRs used in transformation vectors, since a relative amount of *sppv117* transcripts was similar.

However, the P117 and R117 lines differed phenotypically. P117 lines were indistinguishable from wild-type plants, while R117 lines showed signs of stunted growth and a pale green leaf colour due to a decrease in chlorophyll content. The phenotypic changes in the R117 lines were most likely caused by rearrangements of plastid genomes, as demonstrated by Southern blotting. McCabe et al. (2008) linked the yellow phenotype of transplastomic plants expressing human immunodeficiency virus (HIV) p24 antigen obtained with a similar construct to recombination between native and introduced *rbcL* terminators. Plastid promoters, terminators and 5′-UTRs used in transformation vectors duplicate endogenous regulatory signals in chloroplast DNA, which can further cause rearrangements in the plastid genome due to homologous recombination. To avoid unintended recombination events, heterologous sequences with a low level of homology with plastid DNA should be used as regulatory signals in chloroplast vectors (Bock 2014). Another approach to minimising the risk of unintended recombination events was demonstrated by Gray et al. (2008), utilising read-through transcription from native plastid promoters with promoter-free constructs.

The P117 lines expressing the transgene under the control of the *psbA* promoter were phenotypically indistinguishable from wild-type plants; however, the protein yield was relatively low, with only 0.3% of TSP. Further optimisation of the construct is necessary to increase the protein content since, for large-scale production, the protein expression level should preferably exceed 1% of TSP. There are several ways to enhance the protein content: usage of a codon-optimised gene instead of the native gene, transgene integration into the inverted repeat region (IR) of the plastid genome to double the gene copy, or inclusion of a short N-terminal fusion so-called ‘downstream box’ (DB) as a translation enhancer after the translation initiation codon (Bock 2014).

Nevertheless, the achieved SPPV117 accumulation level in this study allows for purifying a sufficient amount of protein for further immunological studies. We have demonstrated that the chloroplast-produced SPPV117 protein retained its antigenicity and according prediction could manifest protective immunity against SPPV.

For the future, we plan to evaluate its potential to induce neutralising antibodies against sheep pox virus.

## Supplementary Information

Below is the link to the electronic supplementary material.

Supplementary file 1 (DOCX 426 kb) Supplementary Figures. This document contains Supplementary figure information.

## Data Availability

The data supporting the results of this article will be provided by the authors upon request.
