# Supplementary material for: Production of the sheep pox virus structural protein SPPV117 in tobacco chloroplasts
Source: Biotechnol Lett. 2021 Apr 2;43(7):1475–85. doi: 10.1007/s10529-021-03117-x (PMC8017516; doi:10.1007/s10529-021-03117-x)
Supplement: Supplementary file 1 — (DOCX 426 kb) Supplementary Figures. This document contains Supplementary figure information. [file 10529_2021_3117_MOESM1_ESM.docx]

**Supplementary Fig. 1** PCR analysis of total DNA from various lines with *sppv117* (**a**) and *aadA* (**b**) gene-specific primers. wt, DNA from wild-type tobacco plants; 1-3, plants transformed with pP117; 4-7, plants transformed with pR117; M, DNA marker; pc_1_, pc_2_, pP117and pR117 plasmids as positive controls, respectively.

**Supplementary Fig. 2** Phenotype of wild-type (wt) and transplastomic plants obtained with transformation vectors pP117 (P117) and pR117 (R117).

**Supplementary Fig. 3** Relative amount of *sppv117* transcripts detected via qPCR. Values are presented as mean ± SD. The level of significance between P117 and R117 lines was 98.36%.

Supplementary **Fig.1**

**
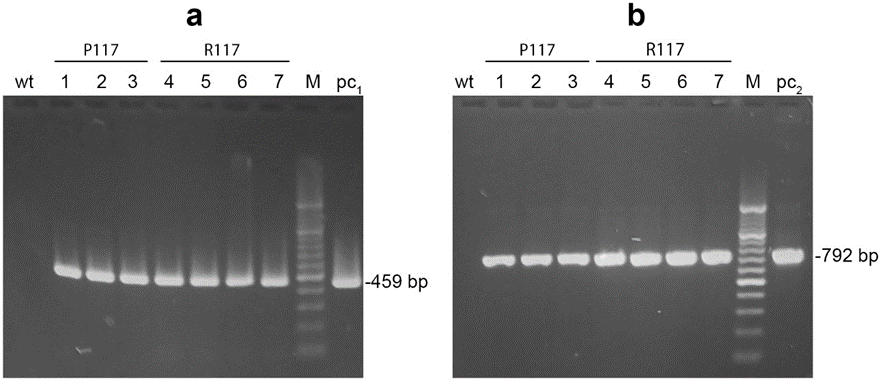
**

Supplementary **Fig. 2**

**
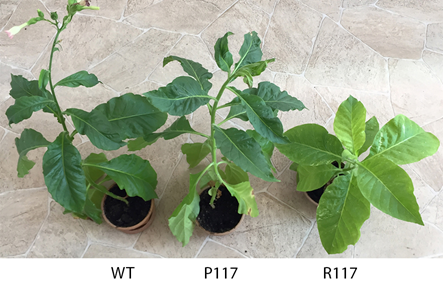
**

Supplementary **Fig. 3**

**
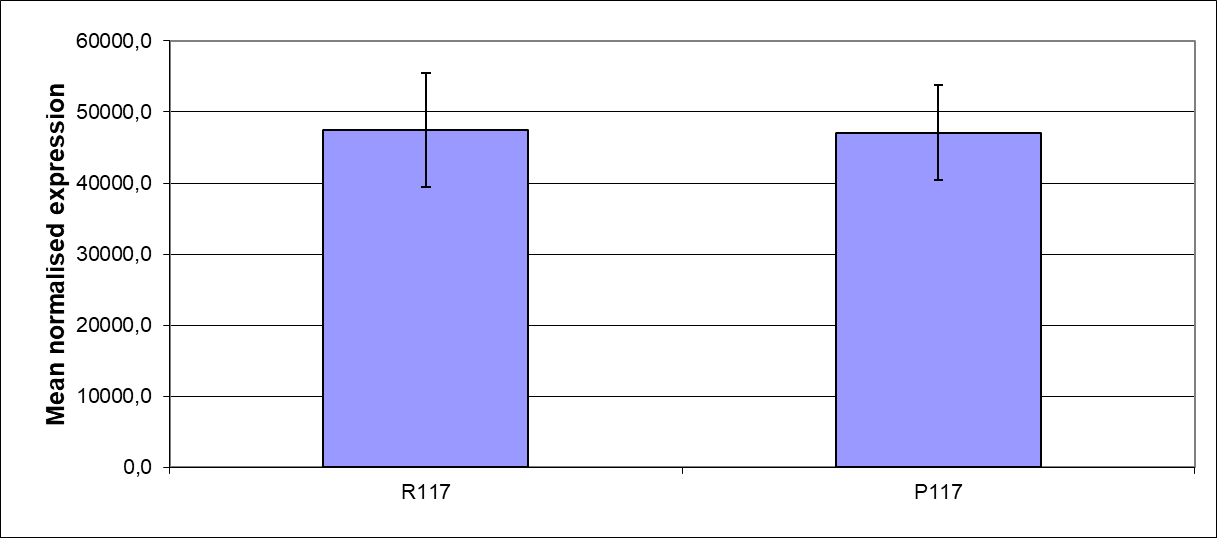
**
